# INDIFURUTO: A novel tool for assessing diabetic foot recurrence risk in type 2 diabetes

**DOI:** 10.25122/jml-2023-0058

**Published:** 2023-10

**Authors:** Haryanto Haryanto, Syahid Amrullah, Suriadi Jais, Supriadi Supriadi, Imran Imran, Yunita Sari

**Affiliations:** 1 Department of Medical Surgical, Wound Management and Critical Nursing, Institut Teknologi dan Kesehatan Muhammadiyah, Kalimantan Barat, Pontianak, Indonesia; 2 Department of Nursing, Faculty of Health Sciences, Universitas Jenderal Soedirman, Purwokerto, Indonesia

**Keywords:** recurrence, diabetic foot ulcers, risk assessment, prediction, detection, prevention

## Abstract

This study aimed to evaluate diabetic foot ulcer recurrence using the Indonesia Diabetic Foot Ulcer Recurrence Assessment Tool (INDIFURUTO), a new diabetic foot risk recurrence assessment tool. This study used a prospective cohort design. A total of thirty-three participants met the inclusion criteria. We used sensitivity, specificity values, AUC, and, respectively, a 95% confidence interval (CI) to calculate prognostic accuracy measures. The results showed that this study had an AUC of 0,97 [95% confidence interval (CI) 0.91-1.00]. The cut-off point (Youden Index) was <45, with sensitivity and specificity values of 100% and 90%, respectively. The utilization of this model can facilitate the monitoring and enhancement of foot ulcer recurrence prevention in individuals diagnosed with diabetes. This study showed that the new model had a high prediction. Therefore, this model better stratifies people at high risk of foot ulceration.

## INTRODUCTION

Diabetes is a collection of metabolic illnesses marked by hyperglycemia induced by insulin secretion, action, or both. Chronic hyperglycemia in diabetes is associated with long-term organ damage, dysfunction, and failure, particularly of the eyes, kidneys, nerves, heart, and blood vessels [1]. According to the International Diabetes Federation (IDF), approximately 463 million individuals were living with diabetes in Indonesia as of 2019. By 2030, that number is projected to rise to 578 million, and by 2045, it is predicted to reach 700 million. Because of this, Indonesia has one of the top ten highest rates of diabetes worldwide [2].

Diabetes often leads to various complications, including diabetic foot ulcers (DFU), a severe consequence characterized by deep tissue lesions in the lower extremities, often accompanied by neurological disorders and peripheral vascular disease [3]. DFUs result from multiple factors, including neuropathies, peripheral arterial disease (PAD), foot deformities, demographic factors (age, sex), duration of diabetes, ethnicity, previous foot issues, and other microvascular complications [4]. A study reported that people with a healed DFU are at increased risk of developing a new foot ulcer, with a recurrence incidence of 33.1% per year [5]. This is supported by a systematic study that reported a high recurrence rate globally [6]. Several risk factors can lead to DFU recurrence [7]. Given the substantial impact of DFUs on quality of life, financial burden, and risks, including amputation and death [8, 9], it is crucial to focus on preventing recurrence.

In Indonesia, the rate of lower extremity amputations among patients with diabetes ranges between 36.3% and 39.5% [10, 11], which is notably higher than in the Netherlands and England (15.5% and 16%, respectively) [12, 13]. Moreover, DFUs severely affect the quality of life and impose significant social and economic burdens due to prolonged healing and high treatment costs [14, 15]. Therefore, assessing the risk of DFU recurrence is necessary to prevent amputations and improve patients' quality of life.

Accurate assessment of the risk of diabetic foot ulcer (DFU) recurrence is also essential for tailoring effective treatment strategies. While numerous classification methods exist for predicting DFU development [16], none specifically address the recurrence of diabetic foot ulcers. To date, no study has evaluated such a risk, particularly in Indonesia. As a result, we aimed to evaluate diabetic foot ulcer recurrence with a new diabetic foot risk recurrent assessment method, INDIFURUTO (Indonesia Diabetic Foot Ulcer Recurrence Assessment Tool) in type 2 diabetes mellitus. In our previous unpublished study, we developed INDIFURUTO through a Delphi method involving an expert panel. This tool demonstrated robust validity, evidenced by a mean authority coefficient of 0.71 and high positive coefficients at 100% and 78%. The Kendall coordination coefficient was statistically significant (χ^2^ test, p<0.01), and the inter-rater reliability agreement was perfect (1.00). Consequently, these findings could assist nurses in predicting diabetic foot ulcer recurrence, potentially improving the quality of life for patients with diabetes.

## MATERIAL AND METHODS

### Research design

This study was conducted as a prospective cohort study. We followed the Standard for Reporting Diagnostic Accuracy (STARD) initiative [17].

### Participants

Participants were selected from a multisite cohort in West Kalimantan, Indonesia, using purposive sampling from July to September 2022. We recruited only individuals who received treatment for type 2 diabetes mellitus (DM) at the Community Health Centre. The study specifically targeted patients who had either experienced a diabetic foot ulcer (DFU) previously or whose initial ulcer had successfully healed throughout a three-month observation period. A total of 33 patients met these criteria and were included in the study. After providing informed consent, participants completed a questionnaire. Inclusion criteria were Indonesian native speakers aged 35 or older and the absence of mental disorders [18, 19].

### Data collection

Data for the new model evaluation included amputation history, smoking, and ankle brachial pressure index (ABPI) value (Table 1). The Ankle Brachial Pressure Index, a key indicator of vascular status in diabetic patients, was measured through a two-step process. The brachial pressure was initially assessed by wrapping the cuff around the patient's upper arm, applying ultrasound gel for better transmission, and confirming signal detection. Once a clear audible signal was established, the cuff was inflated to a pressure 30 mmHg above the point where the pulse signal disappeared and then deflated at 2-3 mmHg per second to identify the systolic pressure.

**Table 1:** Risk factors for recurrence of diabetic foot ulcers

Factors
Amputation history Smoking history Serum glucose level ABPI Monofilament test Skin foot temperature

ABPI (Ankle Brachial Pressure Index)

The measurement of ankle pressure followed a structured approach. The cuff was roughly 2 cm above the malleolus, with the tubes pointing upwards, and pressure was applied to the ankle. The ultrasound gel was applied to the dorsalispedis and posterior tibial arteries to enhance signal detection. The Doppler probe was then methodically angled between 40-60 degrees to pinpoint the optimal signal location. The ABPI was calculated by dividing the lowest value of the dorsalispedis or posterior tibial pressures of the foot by the value of the left or right brachial pressure [20], with values above 1.3 or below 0.9 classified as abnormal (1), and those between 0.8 and 1.0 classified as normal (2) [21].

The monofilament test, an established method for assessing sensory neuropathy, was conducted using a standard Semmes-Weinstein 5.07/10-g monofilament. Eight specific sites of the foot were tested: the plantar aspects of the first, third, and fifth digits; the plantar aspects of the medial, central, and lateral midfoot; the posterior plantar foot; and the interspace between the first and second toes on the dorsal foot surface. Patients who were unable to accurately characterize the location, despite being able to perceive the monofilament, were deemed to have weak test findings [22]. If the patient did not feel the monofilament at any point (less than 8 points), the result was considered negative (value=2). However, the answer was positive (value=1) if the patient felt the monofilament at any one location.

Skin foot temperature was determined based on the difference between the right and left foot temperatures. The present investigation employed the FILR ONE PRO mobile phone external probe infrared thermal imager manufactured by FLIR in the United States. The dimensions of the imager were 68 mm × 34 mm × 14 mm, with a weight of 36.5 g. The device was equipped with both an optical camera and an infrared camera. The mobile device was connected to the FLIR One program through a USB cable to capture images. The available shooting modes encompass visible light images, conventional thermal images, and dynamic enhancement thermal images (MSX). The device could capture static photos, record videos, and create time-lapse sequences. The resolution of visible light can reach up to 1440×1080 dots per inch (dpi), while the thermal resolution can achieve 160×120 dpi. The temperature range spans from -20°C to 400°C, with a resolution of 0.1°C. The mobile device can concurrently exhibit a maximum of three adjustable temperature measurement points and six adjustable temperature measurement areas on its screen. The methodology for monitoring skin foot temperature was derived from the research conducted by Kanazawa [23].

Comprehensive foot care assessment was informed by expert panel guidelines and the International Working Group on the Diabetic Foot (IWGDF), with a Likert scale used to evaluate practices in daily foot checks, physical activity, and knowledge of foot care [24].

1. Daily foot inspection: This area covered five critical practices, including checking the foot daily, touching and feeling its temperature, observing bulla, changing color and shape, studying fingers (dry and fungal), and observing nails.

2. Physical activity: We assessed three aspects of physical activity related to foot health: the execution of at least ten distinct foot-related exercises, the routine performance of these exercises twice daily, and a walking regimen aiming for a minimum of 1,000 steps daily.

3. Knowledge: This domain evaluated the level of the participant’s knowledge in four areas: the recommended foot exercises, walking habits, general foot care practices, and specific strategies for diabetic foot ulcer prevention.

To document the demographic and clinical characteristics of participants, we utilized a standardized data sheet capturing essential information. This included gender, age, occupation, education level, duration of diabetes mellitus (DM), presence of co-morbid conditions, and glycemic control as indicated by Hemoglobin A1c (HbA1c) values. We used the INDIFURUTO rules, a systematic approach based on the scores of specific criteria: history of amputation, smoking history, serum glucose levels, ABPI values, and skin temperature differentials. Each factor was assigned a score of 1 for 'Yes' or 'Abnormal' and 2 for 'No' or 'Normal'.

### Data analysis

We classified the diabetic foot ulcer recurrence risk into three categories: low, medium, and high risk, which were considered clinically relevant. The appropriate cut-off values for these risk classes were determined through a visual examination of the Receiver Operating Characteristic (ROC) curve. By analyzing the curve and the coordinates for sensitivity and specificity, we established the cut-off points that would provide the most clinically relevant separation between the risk categories. The following prognostic accuracy measures were computed: sensitivity, specificity, area under the curve (AUC), and respective 95% confidence intervals (CI). IBM SPSS Statistics for Windows version 26.0 (IBM Corp., Armonk, NY, USA) was used to analyze the data.

## RESULTS

### Participant characteristics

The demographic and clinical characteristics of the participants are detailed in Tables 2 and 3. The study cohort predominantly consisted of female participants (75.8%). The average age of the respondents was 59.2 years±9.5 years and 33.3% had completed junior high school. Most participants (57.6%) had housekeeping roles. The mean duration of DM was 4.8±4.8 years, and hypertension was the most common co-morbid condition, present in 78.8% of participants. The mean serum glucose level was 188.5±91.5 g/dl and the mean HbA1c value was 5.0±4.6%.

**Table 2:** Participants characteristics

Characteristics	Participants (N=33)
**Gender, No (%)** Female Male **Age (years), (Mean±SD)** **Occupation, No (%)** Private Housekeeping Employee Retired **Education, No (%)** No education Elementary school Junior high school Senior high school University **Duration of DM (years), (Mean±SD)** **Co-morbidities, No (%)** No Gastritis Hypercholesterol Hypertension Heart disease Dizziness **Serum glucose level (g/dl), (Mean±SD)** **HbA1c (%), (Mean±SD) (N=32)**	25 (75.8) 8 (24.2) 59.2±9.5 6 (18.2) 19 (57.6) 7 (21.2) 1 (3.0) 2 (6.1) 7 (21.2) 11 (33.3) 7 (21.2) 6 (18.2) 4.8±4.8 1 (3.0) 2 (6.1) 2 (6.1) 26 (78.8) 1 (3.0) 1 (3.0) 188.5±91.5 5.0±4.6

**Table 3:** Risk recurrence categories for diabetic foot ulcers

Categories	Participants (N=33)
High risk	0
Medium risk	24
Low risk	9

High risk (<22), medium risk (23-45), low risk (>46)

### DFU recurrence prediction

**Figure 1:**
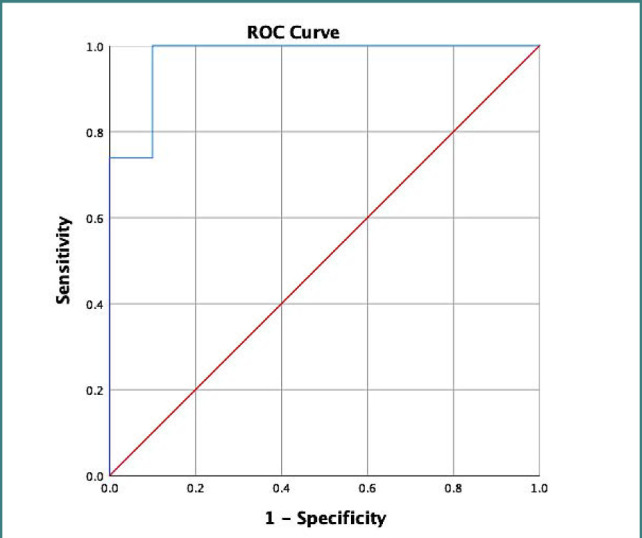
Indonesia Diabetic Foot Ulcer Recurrence Assessment Tool (INDIFURUTO) area under the receiver operating characteristic curve for recurrence prediction. INDIFURUTO classification presents an area under the receiver operating characteristic curve of 97.4% [95% confidence interval (CI) 0.91–1.00]. The cut-off point (Yauden Index) is a score <45 with sensitivity and specificity values of 100% and 90%, respectively.

We classified participants into three risk categories for DFU recurrence using the INDIFURUTO scoring system. According to the system, participants scoring less than or equal to 22 points were categorized as high risk, those scoring between 23 and 45 points were considered medium risk, and those scoring more than or equal to 46 points were classified as low risk, as detailed in Table 3. The area under the curve (AUC) in this study was 0.97 (95% CI: 0.91–1.00) (Figure 1). A score less than 45 with sensitivity and specificity values of 100% and 90%, respectively, was considered the cut-off point (Yauden Index) (Figure 1).

### Discussion

To the best of our knowledge, this study represents the first study on assessing diabetic foot ulcer (DFU) recurrence in Indonesia, utilizing a novel tool for evaluating the risk associated with diabetic foot problems. Our study showed that the INDIFURUTO model had high validity because sensitivity and specificity values were more than 80%, respectively [25]. The clinical relevance of our study is highlighted by categorizing diabetic foot ulcer recurrence risk into three distinct groups: high, moderate, and low risk. This classification supports the approach taken in previous studies. Similarly, a previous study created three risk groups, including low, medium, and high risk [26].

The findings of a prior study, known as the Diabetic Foot Risk Assessment (DIAFORA), showed comparable or superior accuracy in predicting lower extremity amputations among individuals with diabetic foot ulcers [26]. INDIFUROTO system could be utilized to predict the recurrence of diabetic foot ulcers.

The INDIFUROTO model had higher sensitivity and specificity in the present study because we used skin-foot temperature measures in this classification. Consistent with another study, infrared thermography has demonstrated the ability to detect localized temperature variations in individuals with diabetes at increased risk of foot-related complications [27]. Furthermore, another study revealed that the reliability of the thermal imaging system for temperature assessment exhibited a high level of agreement [28]. In addition, previous research has suggested that thermal imaging can serve as an early predictor for the healing of ulcers. Temperature self-assessment may improve the accuracy of this method in predicting the development of foot ulcers in diabetes [29]. Therefore, this model has validity in detecting DFU recurrence.

The current study has several limitations, including a limited sample size. Future studies should aim for a larger and more diverse sample across multiple sites to enhance the generalizability of the findings. One of the strengths of this study is that it is the first in Indonesia to examine DFU recurrence using a novel diabetic foot risk recurrent assessment tool.

## CONCLUSION

The INDIFURUTO model had a high prediction accuracy, demonstrating its effectiveness in stratifying patients according to their risk of developing foot ulcers. The application of the INDIFURUTO model represents a significant advancement in the monitoring and prevention of recurrent foot ulcers in diabetic patients, potentially leading to improved patient outcomes and reduced incidence of complications.

## Personal thanks

The authors would like to thank Kementerian Pendidikan, Kebudayaan, Risetdan Teknologi, Durian Health Center in Kubu Raya, and the nurses of Alianyang Health Center, Pontianak, Kota and Sei for supporting this study.

## References

[1] American Diabetes Association (2010). Diagnosis and classification of diabetes mellitus. Diabetes Care.

[2] International Diabetes Federation (2021). IDF Diabetes Atlas.

[3] Apelqvist J (2012). Diagnostics and treatment of the diabetic foot. Endocrine.

[4] Vowden K (1997). Diabetic foot complications. J Wound Care.

[5] Engberg S, Kirketerp-Møller K, Andersen HU, Rasmussen A (2019). Incidence and predictors of recurrent and other new diabetic foot ulcers: a retrospective cohort study. Diabet Med.

[6] Fu XL, Ding H, Miao W, Mao CX (2019). Global recurrence rates in diabetic foot ulcers: A systematic review and meta-analysis. Diabetes Metab Res Rev.

[7] Huang ZH, Li SQ, Kou Y, Huang L (2019). Risk factors for the recurrence of diabetic foot ulcers among diabetic patients: a meta-analysis. Int Wound J.

[8] Vileikyte L (2001). Diabetic foot ulcers: A quality of life issue. Diabetes Metab Res Rev.

[9] Moulik PK, Tonga MT, Gill GV (2003). Amputation and Mortality in New-Onset Diabetic Foot Ulcers Stratified by Etiology. Diabetes Care.

[10] Decroil E, Karimi J, Manaf A, Syahbuddin S (2008). Profil Ulkus Diabetikpada Penderita Rawat Inap di Bagian Penyakit Dalam RSUP Dr M. Djamil Padang. Maj Kedokt Indon.

[11] Gde T, Pemayun D, Naibaho RM (2017). Clinical profile and outcome of diabetic foot ulcer, a view from tertiary care hospital in Semarang, Indonesia. Diabet Foot Ankle.

[12] Winkley K, Stahl D, Chalder T, Edmonds ME, Ismail K (2007). Risk factors associated with adverse outcomes in a population-based prospective cohort study of people with their first diabetic foot ulcer. J Diabetes Complications.

[13] Peters EJG, Armstrong DG, Lavery LA (2007). Risk Factors for Recurrent Diabetic Foot. Diabetes Care.

[14] Khunkaew S, Fernandez R, Sim J (2019). Health-related quality of life among adults living with diabetic foot ulcers: a meta-analysis. Qual Life Res.

[15] Huidi T, Pauline K, Lucien L, Mukisi-M M (2018). Cost of diabetic foot in France, Spain, Italy, Germany and United Kingdom: A systematic review. Ann Endocrinol (Paris).

[16] Monteiro-Soares M, Boyko EJ, Jeffcoate W, Mills JL (2020). Diabetic foot ulcer classifications: A critical review. Diabetes Metab Res Rev.

[17] Bossuyt PM, Reitsma JB, Bruns DE, Gatsonis CA (2003). Towards complete and accurate reporting of studies of diagnostic accuracy: the STARD initiative. BMJ.

[18] Sari Y, Purnawan I, Taufik A, Sumeru A (2018). Quality of Life and Associated Factors in Indonesian Diabetic Patients with Foot Ulcers. Nurse Media J Nurs.

[19] Sulistyo Hadi AA, Sia WS, Maneewat K (2018). The effect of a foot care camp on diabetic foot care knowledge and the behaviours of individuals with diabetes mellitus. J Res Nurs.

[20] Schaper NC, van Netten JJ, Apelqvist J, Bus SA (2020). Practical Guidelines on the prevention and management of diabetic foot disease (IWGDF 2019 update). Diabetes Metab Res Rev.

[21] Al-Qaisi M, Nott DM, King DH, Kaddoura S (2009). Ankle Brachial Pressure Index (ABPI): An update for practitioners. Vasc Health Risk Manag.

[22] Vural S, Bostanci S, Koçyigit P, Çaliskan D (2018). Risk Factors and Frequency of Ingrown Nails in Adult Diabetic Patients. J Foot Ankle Surg.

[23] Kanazawa T, Nakagami G, Goto T, Noguchi H (2016). Use of smartphone attached mobile thermography assessing subclinical inflammation: a pilot study. J Wound Care.

[24] Bus SA, Lavery LA, Monteiro-Soares M, Rasmussen A (2020). Guidelines on the prevention of foot ulcers in persons with diabetes (IWGDF 2019 update). Diabetes Metab Res Rev.

[25] Deeks JJ (2001). Systematic reviews of evaluations of diagnostic and screening tests. BMJ.

[26] Monteiro-Soares M, Dinis-Ribeiro M (2016). A new diabetic foot risk assessment tool: DIAFORA. Diabetes Metab Res Rev.

[27] Ilo A, Romsi P, Mäkelä J (2020). Infrared Thermography and Vascular Disorders in Diabetic Feet. J Diabetes Sci Technol.

[28] Petrova NL, Whittam A, MacDonald A, Ainarkar S (2018). Reliability of a novel thermal imaging system for temperature assessment of healthy feet. J Foot Ankle Res.

[29] Petrova NL, Donaldson NK, Tang W, MacDonald A (2020). Infrared thermography and ulcer prevention in the high-risk diabetic foot: data from a single-blind multicentre controlled clinical trial. Diabet Med.

